# Supplementary material for: Intra- and Interspecies Conjugal Transfer of Plasmids in Gram-Negative Bacteria
Source: Biomedicines. 2025 Jan 20;13(1):238. doi: 10.3390/biomedicines13010238 (PMC11760439; doi:10.3390/biomedicines13010238)
Supplement: Supplementary file 1 [file biomedicines-13-00238-s001.zip › biomedicines-3426488-supplementary/Supplementary figures.pdf]

Supplementary figures

Intra- and Interspecies conjugal transfer of plasmids in Gram-negative bacteria

Julia R. Savelieva, Daria A. Kondratieva and Maria V. Golikova

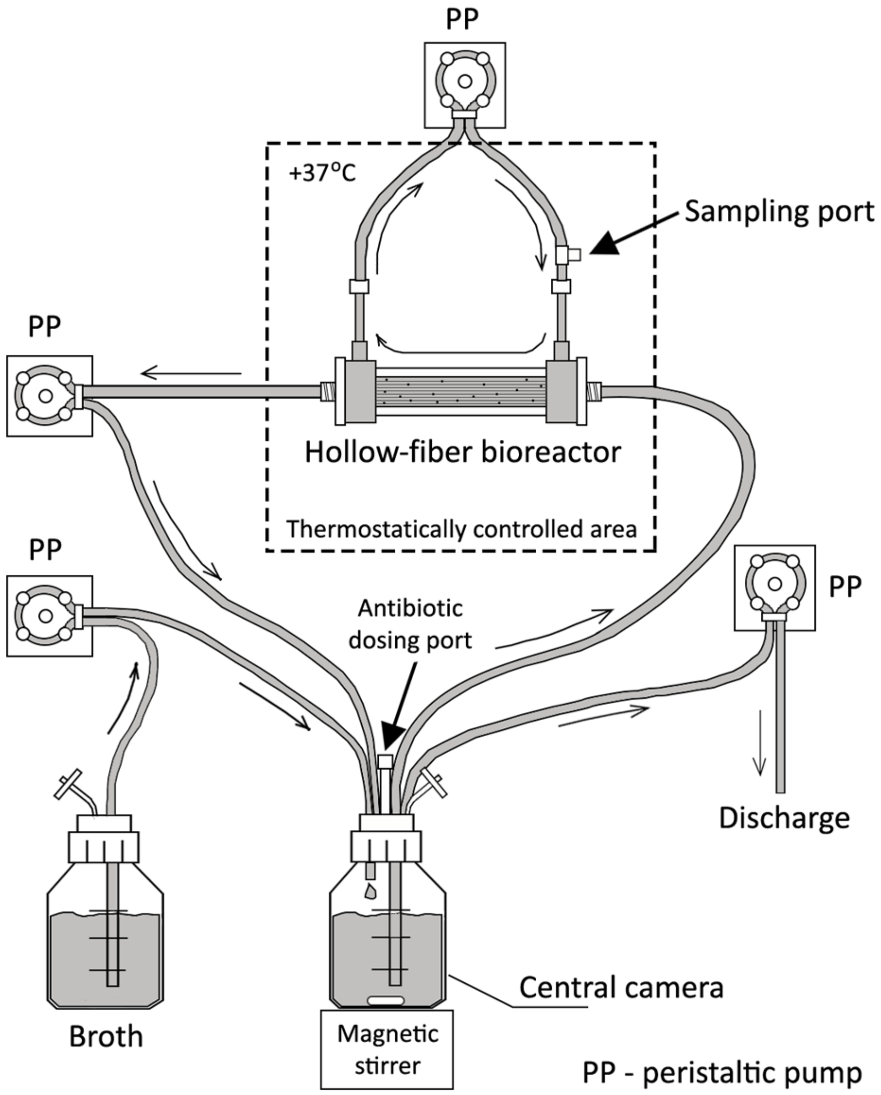

Figure S1. A schematic illustration of the hollow-fiber infection model (HFIM).

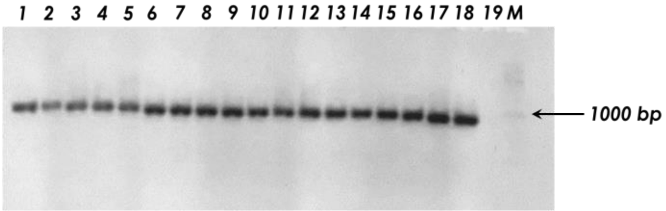

Lines 1-4 - K. pneumoniae 700603/565 transconjugants
Lines 5-8 - K. pneumoniae 188/565 transconjugants
Lines 9-12 - K. pneumoniae 700603/485 transconjugants
Lines 13-16 - K. pneumoniae 188/485 transconjugants
Lines 17, 18 – donor K. pneumoniae 565 and 485 (positive control)
Line 19 – recipient K. pneumoniae ATCC 700603 (negative control)
M – DNA marker (250-10000 bp)

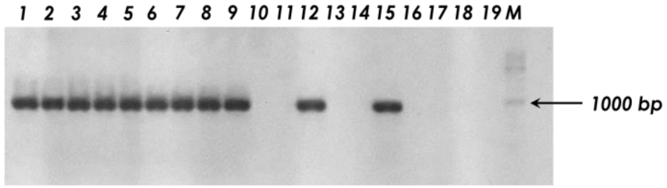

Lines 1-4 - K. pneumoniae 700603/38 transconjugants
Lines 5-8 - K. pneumoniae 188/38 transconjugants
Line 9 - donor K. pneumoniae 38 (positive control)
Lines 10-13 – E. coli C600/565 transconjugants
Lines 14-17 – E. coli 25922/565 transconjugants
Lines 18, 19 – recipient E. coli C600 and ATCC 25922 (negative control)
M – DNA marker (250-10000 bp)

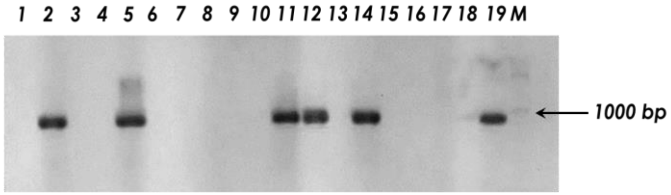

Lines 1-4 - E. coli C600/485 transconjugants
Lines 5-8 - E. coli 25922/485 transconjugants
Lines 9-12 - E. coli C600/38 transconjugants
Lines 13-16 – E. coli 25922/38 transconjugants
Lines 17, 18 – recipient E. coli C600 and ATCC 25922 (negative control)
Line 19 – donor K. pneumoniae 485 (positive control)
M – DNA marker (250-10000 bp)

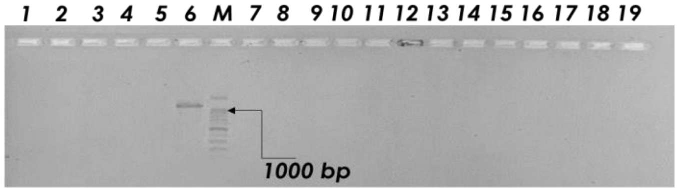

Lines 1-5 - P. aeruginosa 9027/38 transconjugants
Lines 6 - donor K. pneumoniae 38 (positive control)
Lines 7-11 - P. aeruginosa 27853/38 transconjugants
Lines 13, 14 – recipient P. aeruginosa ATCC 9027 and ATCC 27853 (negative control)
Lines 15-19 - P. aeruginosa 9027/38 transconjugants
M – DNA marker (100-1500 bp)

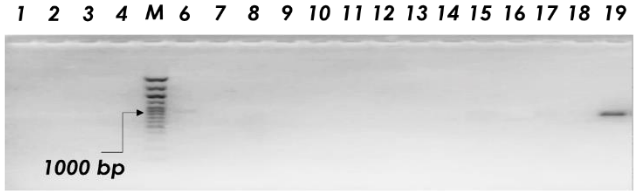

Lines 1-4 - P. aeruginosa 9027/565 transconjugants
Lines 6-9 - P. aeruginosa 27853/565 transconjugants
Lines 10-13 - P. aeruginosa 9027/485 transconjugants
Lines 14-17 – P. aeruginosa 27853/485 transconjugants
Line 18 – recipient P. aeruginosa ATCC 9027 (negative control)
Line 19 – donor K. pneumoniae 565 (positive control)
M – DNA marker (250-10000 bp)

Figure S2. Agarose gel electrophoresis of *K. pneumoniae*, *E. coli* and *P. aeruginosa* transconjugants amplification products, 10V/cm, 0.9% agarose, DNA size markers 250-10000 bp or 100-1500 bp.

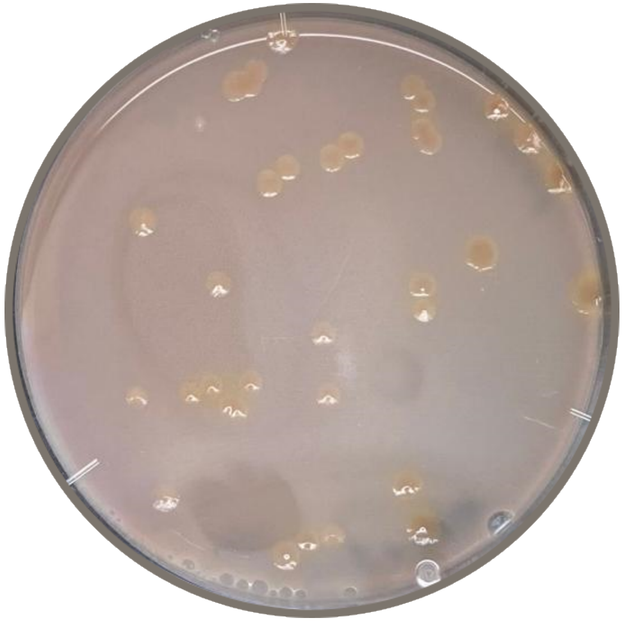

Figure S3. “Possible” transconjugant colonies of *P. aeruginosa* grown on agar with rifampicin and meropenem.
