# Supplementary material for: Intra- and Interspecies Conjugal Transfer of Plasmids in Gram-Negative Bacteria
Source: Biomedicines. 2025 Jan 20;13(1):238. doi: 10.3390/biomedicines13010238 (PMC11760439; doi:10.3390/biomedicines13010238)
Supplement: Supplementary file 1 [file biomedicines-13-00238-s001.zip › biomedicines-3426488-supplementary/Supplementary tables.pdf]

Supplementary tables

Intra- and Interspecies conjugal transfer of plasmids in Gram-negative bacteria

Julia R. Savelieva, Daria A. Kondratieva and Maria V. Golikova

Table S1. List of PCR primer used for the experiments.

Genes	Forward primer	Reverse primer
MobA (pOXAAPSS)	TGCTCACGAATCAAACG	AATAAACGTCTGCAGCG
RepA (pOXAAPSS)	CTTTCAGCTTCTTCTCG	TGATATGTTAAGCGGCG
TraI (pKPCAPSS)	GTCGTTTTCTCCGTACC	AGGTCTACACCGACAAC
RepA (pKPCAPSS)	CCAGTCGCGTGTAGTTG	TCACCCATTACCGGCAG
bla_{KPC-2}	CGTCTAGTTCTGCTGTC	TGTGCTTGTCATCCTTG
bla_{OXA-48}	ATTAGCCTTATCGGCTG	TCCTGTTTGAGCACTTC

Table S2. MIC data (in µg/ml) of recipients and selected transconjugants following *in vitro* mating with donor *K. pneumoniae* 565.

**Antibiotic**	***K. pneumoniae* ATCC 700603**	***K. pneumoniae* 700603/565**	***K. pneumoniae* 188**	***K. pneumoniae* 188/565**	***E. coli* C600**	***E. coli* C600/565**	***E. coli* ATCC 25922**	***E. coli* 25922/565**
Meropenem	**0.06**	**4**	**0.03**	**4**	**0.03**	**2**	**0.03**	**1**
Ciprofloxacin	**0.25**	**4**	>16	>16	**0.25**	**2**	**4**	**16**
Doxycycline	8	>16	>16	>16	1	1	2	2
Azithromycin	>16	>16	>16	>16	**4**	**>16**	**4**	**>16**
Polymyxin B	0.5	0.5	0.5	0.5	0.125	0.125	0.125	0.125
Ceftazidime	>16	>16	>16	>16	**0.25**	**>16**	**1**	**>16**
Gentamicin	4	4	0.5	0.5	0.5	0.25	0.125	0.125
Chloramphenicol	>16	>16	>16	>16	4	4	>16	>16
Ampicillin	>16	>16	>16	>16	8	>16	8	>16
Piperacillin/ Tazobactam	8	>16	>16	>16	**2**	**>16**	**2**	**>16**

Bold indicates values that differ by more than two-fold.

Table S3. MIC data (in μg/ml) of recipients and selected transconjugants following *in vitro* mating with donor *K. pneumoniae* 485.

**Antibiotic**	***K. pneumoniae*** **ATCC 700603**	***K. pneumoniae*** **700603/485**	***K. pneumoniae*** **188**	***K. pneumoniae*** **188/485**	***E. coli*** **C600**	***E. coli*** **C600/485**	***E. coli*** **ATCC 25922**	***E. coli*** **25922/485**
Meropenem	**0.06**	**0.5**	**0.03**	**0.25**	**0.03**	**0.5**	**0.03**	**0.5**
Ciprofloxacin	0.25	0.25	>16	>16	0.25	0.25	4	4
Doxycycline	8	8	>16	>16	1	1	2	2
Azithromycin	>16	>16	>16	>16	4	4	4	4
Polymyxin B	0.5	0.5	0.5	0.5	0.125	0.125	0.125	0.125
Ceftazidime	>16	>16	>16	>16	0.25	0.25	1	1
Gentamicin	4	4	0.5	0.5	0.5	0.5	0.125	0.125
Chloramphenicol	>16	>16	>16	>16	4	4	>16	>16
Ampicillin	>16	>16	>16	>16	8	>16	8	>16
Piperacillin/ Tazobactam	8	>16	>16	>16	**2**	**>16**	**2**	**>16**

Bold indicates values that differ by more than two-fold.

Table S4. MIC data (in μg/ml) of recipients and selected transconjugants following *in vitro* mating with donor *K. pneumoniae* 38.

**Antibiotic**	***K. pneumoniae*** **ATCC 700603**	***K. pneumoniae*** **700603/38**	***K. pneumoniae*** **188**	***K. pneumoniae*** **188/38**	***E. coli*** **C600**	***E. coli*** **C600/38**	***E. coli*** **ATCC 25922**	***E. coli*** **25922/38**
Meropenem	**0.06**	**0.5**	**0.03**	**0.5**	**0.03**	**0.25**	**0.03**	**0.5**
Ciprofloxacin	0.25	0.25	>16	>16	0.25	0.25	4	4
Doxycycline	8	8	>16	>16	1	1	2	2
Azithromycin	>16	>16	>16	>16	4	4	4	4
Polymyxin B	0.5	0.5	0.5	0.5	0.125	0.125	0.125	0.125
Ceftazidime	>16	>16	>16	>16	0.25	0.25	1	1
Gentamicin	4	4	0.5	0.5	0.5	0.5	0.125	0.125
Chloramphenicol	>16	>16	>16	>16	4	4	>16	>16
Ampicillin	>16	>16	>16	>16	8	>16	8	8
Piperacillin/ Tazobactam	8	>16	>16	>16	2	**>16**	2	**>16**

Bold indicates values that differ by more than two-fold.

Table S5. Conjugation frequencies between donor *K. pneumoniae* and recipient *K. pneumoniae*, *E. coli* and *P. aeruginosa* strains. All data are represented as arithmetic mean ± standard error of the mean.

**Donor strains**	**Recipient strains**					
	E. coli		***K. pneumoniae***		***P. aeruginosa***	
	**C600**	**ATCC 25922**	**ATCC 700603**	**188**	**ATCC 9027**	**ATCC 279853**
K. pneumoniae 38	$3.27 \pm 0.53 \times 10^{-7}$	$2.50 \pm 0.49 \times 10^{-7}$	$8.03 \pm 2.21 \times 10^{-6}$	$4.17 \pm 1.01 \times 10^{-7}$	$\mathbf{1 \times 10^{-9}}$	$\mathbf{1 \times 10^{-9}}$
K. pneumoniae 485	$1.72 \pm 0.36 \times 10^{-6}$	$5.00 \pm 0.78 \times 10^{-6}$	$1.79 \pm 0.4 \times 10^{-4}$	$4.81 \pm 1.07 \times 10^{-5}$	$\mathbf{1 \times 10^{-9}}$	$\mathbf{1 \times 10^{-9}}$
K. pneumoniae 565	$1.19 \pm 0.19 \times 10^{-4}$	$1.20 \pm 0.23 \times 10^{-5}$	$2.24 \pm 0.51 \times 10^{-3}$	$1.20 \pm 0.24 \times 10^{-4}$	$\mathbf{1 \times 10^{-9}}$	$\mathbf{1 \times 10^{-9}}$

The data corresponding to the absence of conjugation as confirmed by PCR is depicted as equivalent to a limit of detection for conjugation frequency of 10^{-9}, and is highlighted in **bold**.
